# Distribution Patterns of Microplastics Pollution in Urban Fresh Waters: A Case Study of Rivers in Chengdu, China

**DOI:** 10.3390/ijerph19158972

**Published:** 2022-07-23

**Authors:** Juan Chen, Yinger Deng, Yong Chen, Xin Peng, Han Qin, Tao Wang, Chenchen Zhao

**Affiliations:** 1 College of Environment and Civil Engineering, Chengdu University of Technology, Chengdu 610059, China; chenjuan310@163.com; 2 Chengdu Environmental Monitoring Center Station of Sichuan Province, Chengdu 610066, China; linmuxi2022@163.com (Y.C.); hancock121212@126.com (H.Q.); wt263691764@163.com (T.W.); zcc202206@126.com (C.Z.); 3 Sichuan Provincial Engineering Research Center of City Solid Waste Energy and Building Materials Conversion and Utilization Technology, Chengdu University, Chengdu 610106, China; pengxin@cdu.edu.cn

**Keywords:** microplastics, Chengdu, urban rivers, surface water, pollution

## Abstract

Microplastics are widely found in oceans and rivers. In China, the research on microplastic pollution in inland urban fresh waters of China is insufficient. We studied microplastics in the surface waters of urban rivers in Chengdu, which is the largest city in western China. The concentration of microplastics in the analysis environment ranged from 5.00 to 10.5 items/L, and the average quantity was 8.82 items/L. The majority of the microplastics were transparent and took the form of fragments, particles, and fibers. Polyethylene terephthalate (PET) and polyamide (PA) were the dominant polymer types of the microplastics analyzed. Plastic particles ≤ 500 μm accounted for 69.8% of the total. This large proportion of small transparent microplastics in urban rivers in Chengdu is a potential threat to the growth of aquatic organisms and birds foraging from the river and may pose hazards to human health. Additionally, the correlation of microplastic content with population quantity and economic level was calculated by the Pearson coefficient method (*p* < 0.05), and the results showed that both have an important effect on the number of microplastics in rivers. This research provides a reference for understanding the level of microplastics in urban rivers in Chengdu and pollution control.

## 1. Introduction

The invention of plastic has resulted in many conveniences in people’s lives. However, the adverse impact that plastic has on the ecological environment is also of increasing concern [1,2]. In 2018, nearly 360 million tons of plastic were produced worldwide, and approximately 25% of the waste produced after plastic consumption could not be recycled [3]. Through physical, chemical, biological, and other functions, the abandoned plastics are ultimately decomposed into tiny plastic particles in nature [4]. Microplastics are defined as small plastics < 5 mm in size [5,6]. The source of microplastics is not only the fragmentation of large volumes of plastic but also the production of tiny particles of plastic, which are often found in facial cleansers, body washes, and toothpaste, etc. Common types of microplastics include polyethylene (PE), polypropylene (PP), polystyrene (PS), polyurethane (PU), polyamide (PA), polyvinyl chloride (PVC) and polyethylene terephthalate (PET) [7]. Although microplastic particles are small, when discharged into the environment, their harmful effects cannot be ignored [8]. In recent years, microplastics have attracted extensive attention as a new type of pollutant [4,9,10]. Plastic particles in aquatic ecological environments are readily ingested by aquatic organisms, accumulate in the body, and pass through the food web, thus threatening human health [11]. Moreover, microplastics can absorb heavy metals and persistent organic pollutants and become carriers of these pollutants [12,13]. In addition, some chemicals are added to the plastic production process to change and increase its functionality, such as plasticizers, antioxidants, dyes, and colors. Continuous leaching of these additives in the environment leads to an increase in ambient concentration and harms the ecological environment [14].

The microplastics in the ocean mainly come from the input of land-based plastics, while rivers are considered to be the transport channels for both the plastic granules in the terrestrial environment and in the ocean [15,16]. Microplastics have been found in rivers in Germany [17], Britain [18], Canada [19], and China [20] in varying abundance. Moore et al. [21] monitored the microplastics in the surface water of two rivers in southern California. The Saigon River in Ho Chi Minh City, Vietnam, contains hundreds of microplastics per liter of surface water [22]. Notably, urban rivers that are more affected by human activities tend to show higher levels of microplastics and pollution [23], indicating that they are also facing more severe microplastic pollution. However, compared with a large number of studies on microplastics in the ocean, research on microplastic pollution in urban rivers is still lacking. In 2020, the Chinese government issued the Opinions on Further Strengthening the Control of Plastic Pollution [24]. It is necessary to enhance research on the mechanisms, monitoring, and prevention and control technology and policy of plastic waste and microplastic pollution in rivers, lakes, and seas, and carry out ecology environmental impacts and human health risk assessments. Consequently, it is essential to investigate the type, size, and distribution of microplastics in rivers, which can provide a scientific basis for the subsequent management of microplastic pollution. Existing studies in the literature show that microplastic pollution in urban rivers in China is mainly concentrated in coastal and central areas [16,20,23], but there is little research on western rivers.

Chengdu is the capital of Sichuan Province and the largest city in western China, with an urban population of more than 20 million and an average population density above 10,000 people/km^2^. Chengdu is located in the upper reaches of the Yangtze River, and the middle reaches of the Minjiang River (an important tributary of the Yangtze River) passes through Chengdu city, which is rich in water resources. The high-density populations, large amounts of sewage discharge, and extensive use of plastic products brought about a mass of pollutant emissions. All these factors create a very high risk of microplastic pollution. Therefore, in this paper, the abundance, size, color, shape, and polymer types of microplastics in the surface water of the Minjiang River flowing through the Fuhe, Shahe, Nanhe, and Jinma Rivers in Chengdu city were determined. On this basis, the pollution status and behavior of microplastics in urban rivers in Chengdu were further discussed.

## 2. Materials and Methods

### 2.1. Study Area and Sample Collection

**Figure 1:**
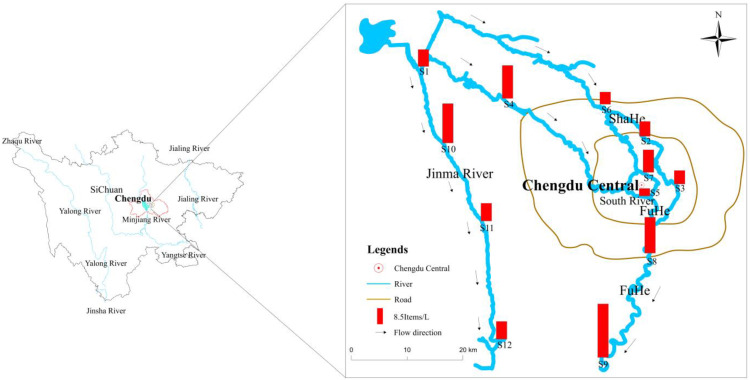
The geographic positions of the sample sites and the distribution of cities.

The Minjiang River in Chengdu is about 100 km long, with a catchment covering an area of approximately 30,000 km^2^. The main rivers in the study area after the Minjiang River enters Chengdu include the Jinma River, Fuhe, Shahe, and South River, which are taken as the research objects (Figure 1). A total of 12 sampling points were set up in this study. Sample points were positioned in areas with straight river sections, steady riverbeds, smooth water flows, and wide water surfaces, while avoiding dead water areas, backwater areas, sewage outlets, and areas with rapids or shoals. Additionally, the industrial development level, population density, and other factors were also considered. Sampling point S1 was situated in Dujiangyan, where the Minjiang River enters Chengdu city, and this point is the control point of this study. Sampling sites S2~S3 were located in Shahe (length: 22 km), S4~S5 in South River (length: 37 km), S6~S9 in Fuhe (length: 115 km), and S10~S12 in the Jinma River (length: 81 km). Sample points S2, S4, and S6 were located upstream of the central urban area or industrial park, while S3, S5, and S7 were positioned upstream of the confluence with the Fuhe. At sampling site S8, the South River and Shahe merge with the Fuhe. These three rivers account for 14% of the city’s drainage area but represent approximately 45% of the population and nearly 80% of Gross Domestic Product (GDP) Chengdu has a subtropical humid monsoon climate with a mild climate and abundant precipitation. About 50% of the annual rainfall occurs from June to August. Although rainwater is frequent in autumn, the total precipitation amount is not large and the water flow changes little, and it is relatively stable. In September 2021, water samples were collected at 0.5 m below the surface at the center of the river with a glass water extractor by boat. After collection, the samples were placed in clean glass bottles and transported back to the laboratory as soon as possible. Before analysis, the samples were stored in a refrigerator at 4 °C.

### 2.2. Laboratory Analysis

A vacuum pump was used to extract and filter the sample onto 0.45 μm glass filter paper. The filter paper was placed into a clean glass Petri dish with a lid with toothless tweezers. Then, 35% H_2_O_2_ [25] was added to remove organic matter and microorganisms from the microplastics. After approximately 48 h, a vacuum pump was used to drain the solution in the Petri dish, and the initial filter paper and the Petri dish were repeatedly washed with deionized water. All flushing liquid was pumped out, and then the wall of the extractor was repeatedly washed with deionized water so that all the target objects were concentrated on the filter paper. Toothless tweezers were used to place the filter paper in a clean glass petri dish with a lid, and it was dried in an oven at low temperature (40~50 °C). After drying, the samples on the filter membrane were carefully transferred to a gold mirror, and the surface characteristics of the microplastics were observed with a stereomicroscope (SC-III; Shanghai Yixiang Instrument Co., Ltd.; Shanghai, China). Then, the surface characteristics of the microplastics were observed by scanning electron microscopy (SEM) (Prisma E; Thermo Fisher Scientific; Waltham, MA, USA). Subsequently, the main types of functional groups in the qualitative samples were characterized by a microscopic infrared spectrometer (Nicolet In10 MX; Thermo Fisher Scientific; Waltham, MA, USA) [26]. The obtained infrared spectra were matched with the standard spectra by similarity matching, and the types, sizes, and numbers of polymers were counted.

To ensure the accuracy of the data, the use of plastic products was avoided in the sampling and experimental analysis process. In this study, the concentration of microplastics in surface water was measured as “items/L”.

## 3. Results and Discussion

### 3.1. The Abundance and Distribution of Microplastics

Microplastics were detected in all river surface water samples. The concentrations of microplastics in the urban rivers varied from 5.00 to 10.5 items/L, which was lower than those of Taihu Lake, at 3.4~25.8 items/L [27], and the Los Angeles River, at 12.9 items/L [15], and slightly higher than those in urban rivers in Wuhan, China 0.921~10.516 items/L [23]. Chengdu and Wuhan are both new first-tier cities in China. Compared to Chengdu, Wuhan has a wider area of water, accounting for almost a quarter of the city’s area, but nearly 8 million fewer people. Therefore, it is reasonable for the microplastic content in urban rivers to be slightly higher than that in Wuhan. Of the four urban rivers, the concentration of microplastics in the Shahe was the lowest with an average of 5.00 items/L. Second, the concentration in the South River was 7.75 items/L. The quantity in Fuhe was the highest, with an average of 10.5 items/L. The number of S9 in Fuhe was as high as 17.0 items/L. The reasons may be as follows: first, the South River and Shahe flow into the Fuhe, increasing pollution sources; second, the Fuhe flows through Chengdu Shuangliu District and the Chengdu Hi-Tech Industrial Development Zone, both of which have populations of more than one million and are areas with relatively good economic development. The large population and high degree of industrial economic development may increase the pollution of the water ecological environment. The average concentration of microplastics in the Jinma River is 9.83 items/L, which is slightly lower than that in the Fuhe. It is speculated that the distance between the Jinma River and the urban center is greater than that between the Fuhe River and the urban center, and the population density is relatively low, which is similar to the research results of Battulga et al. [21] Sampling site S1, with a concentration of 6.50 items/L, is located in Dujiangyan and used as a control spot in this study after the Minjiang River passes through Chengdu city. Before reaching Dujiangyan and entering the Chengdu Plain, the Minjiang River flows through areas with a low population density, low industrial development level, and good ecological environments. Therefore, the sources of microplastic pollution may be mainly domestic sewage discharge and surface runoff pollution along the coast. In general, the microplastic contents of urban rivers in Chengdu range from medium to low levels (Table 1), indicating potential pollution risks.

In an aquatic environment, many factors can contribute to the distribution and content of microplastics, such as the plastic characteristics, hydrogeological environment, meteorological environment [28], surrounding urban population density, and industrial maturity [22,23]. Some microbes attach to the surface of plastic particles, and these changes in weight or properties can also impact their distribution [29] Therefore, these may be the reason for the difference in microplastic concentrations in city rivers (Figure 1). To understand the relationship between urban development and microplastics content, we determined the population of the area through which the river passes and the GDP of the area in the first half of 2021 [30,31,32] and applied the Pearson correlation coefficient method to analyze the correlation between the average concentration of microplastics in each river and these factors (Table 2) (*p* < 0.05). The microplastic content in studied rivers was significantly correlated with the population size. Although there was some degree of relationship with economic development, the correlation was not strong. A high population and human activities produce a large amount of plastic waste in a city, and some plastic items are even directly discharged into the aquatic system, resulting in higher microplastic levels in the surface water of rivers in densely populated areas. For example, the surface water of Beihu lake, Wuhan, China, the lake area closest to the city center, was found to have the highest quantities of microplastics [22]. Battulg et al.’s study also showed that the total number of plastics is always higher on the river shore close to the urban center. Consequently, population density and economic development are significant factors affecting the distribution of microplastics in an aqueous environment.

### 3.2. Morphological Characteristics of Microplastics

**Figure 2:**
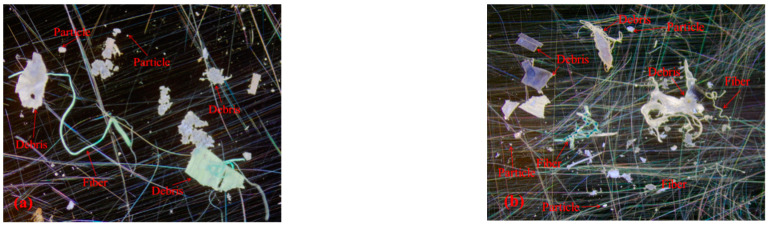
The morphology of the microplastics under a stereomicroscope (magnification: 20×~40×). (**a**,**b**) were from water samples in the study area.

**Figure 3:**
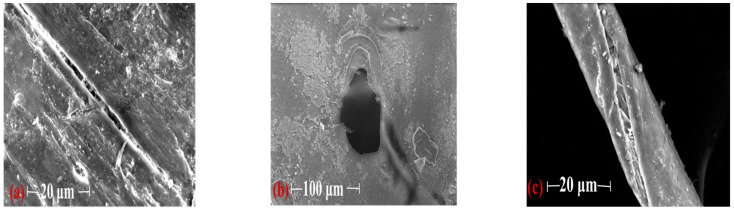
Photographs of selected microplastics (particle (**a**), debris (**b**), fiber (**c**)) under SEM. (**a**–**c**) were chosen under a stereomicroscope.

**Figure 4:**
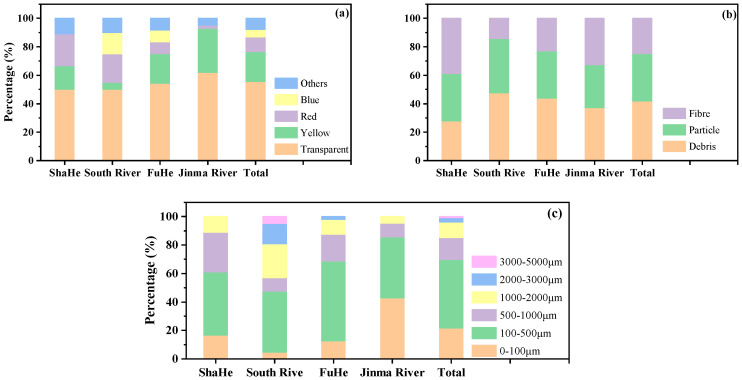
The distribution of microplastics in terms of color (**a**), morphology (**b**), and categories (**c**) in the rivers.

Microplastics often exist in the water environment in the form of fibers, particles, debris, and spheres [13]. Under a stereomicroscope, the microplastics from the urban rivers are mostly transparent, red, yellow, blue, and other colors (Figure 2), and mainly exist in the form of particles, debris, and fibers (Figure 2). To further observe the morphological characteristics of the plastic granules, some were selected using a stereomicroscope and observed under a scanning electron microscope (SEM). The SEM shows that algae, residue, and other substances are attached to the surface of the microplastics. Cracks or holes in the interior indicate that microplastics are degrading (Figure 3a,b). However, less material was attached to the surfaces of the fibers, and the curved surfaces may not be conducive to fixation (Figure 3c). Transparent microplastic accounted for more than half of the rivers in the research region, and the proportions of microplastic in three forms of particles, fragments and fibers were almost the same (Figure 4a,b). Based on their size, we classify microplastics into six levels: I: 0~100 μm, II: 100~500 μm, III: 500~1000 μm, IV: 1000~2000 μm, V: 2000~3000 μm, and VI: 3000~5000 μm. The plastic particles in the urban rivers in Chengdu were small in size, mainly grade Ⅱ, followed by grade I, which together account for 69.8% of the total microplastics. The proportion of microplastics of these two sizes in the Jinma River is as high as 85.8% (Figure 4c). The smaller the microplastic particles, especially the transparent ones, the more likely they are to be eaten by aquatic organisms. Jiana Li et al. [9] studied the microplastic content in bivalve molluscs in water bodies and found that the number of microplastics in scapharca subcrenata was as high as 10.5 items/L, in which items smaller than 250 μm accounted for 33–84% of the total. Small transparent plastics granules account for more than 50% of the content of microplastics in the urban rivers in Chengdu, and most were grade I and II; this kind of microplastic can easily be swallowed by aquatic organisms and birds living around the river banks, thus affecting their growth. Microplastics that become concentrated in organisms may also pose a potential threat to human health as they travel through the food web.

### 3.3. Type Identification of Microplastics

**Figure 5:**
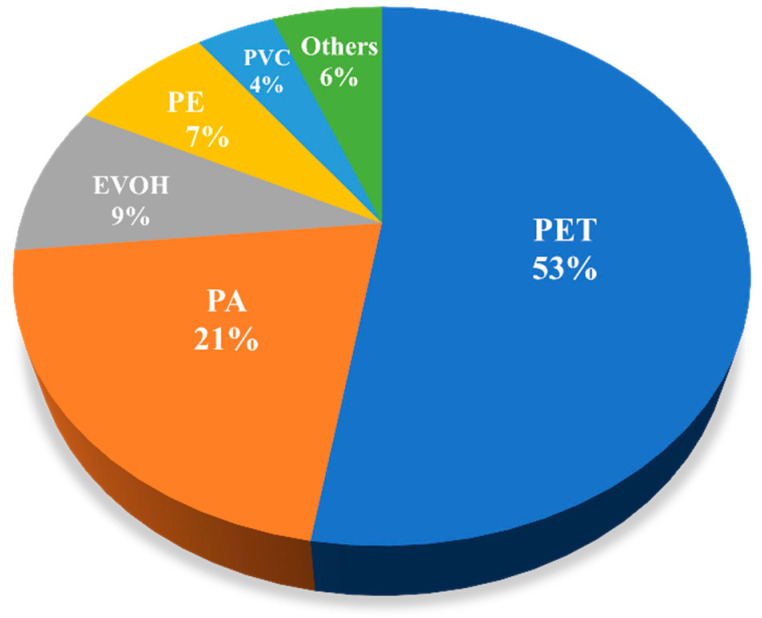
Types of polymers in microplastics in studied rivers.

The functional groups of microplastics were characterized by Fourier transform infrared spectroscopy (FTIR). FTIR has the advantage of simple operation and accurate identification results [26]. In this study, the microplastic functional groups in all samples were characterized by a microscopic infrared spectrometer. The main types of microplastics in the urban river in Chengdu were PET and PA, which accounted for 52.5% and 20.8% of the total microplastics in all samples, respectively. Ethylene vinyl alcohol (EVOH), PE, PVC, and other types were also present (Figure 5). Wang et al. [22] identified 44 microplastic particles in urban rivers in Wuhan and noted that PET was the main type of microplastics, accounting for 40.9%. Lei et al. [33] found that the main type of microplastics in rivers and ports in Melbourne, Australia, is PA. In addition, PE [16,22] and PS [23] are common types of microplastics in some urban rivers. Many baby bottles, tableware, mineral water bottles, and so on are made of PET materials. PET is also widely used in the packaging industry, electronic appliances, medical and health care, construction, automobiles, environmental landscapes, and other fields. PA is mainly used as a synthetic fiber in clothing, tires, mechanical equipment parts, and so on. EVOH is an excellent material for blocking gas, odor, flavor, and solvents, so it is widely used in food packaging materials, medicine, health products, cosmetics, automobile fuel tanks, and other fields. PE has strong plasticity and a low price and is often made into film products for packaging, cable wrapping layers, and household necessities. PVC is also a popular synthetic material. Many decorative advertising cards, exhaust pipes, toilet water tanks, and so on are made of PVC. Although the use of plastic products can be convenient, it is worth noting that some organic and inorganic ingredients are added in the production process of plastic to change its properties [34], and these substances are released into the environment under certain conditions, harming the environment and even human health. For example, at high temperatures or under long-term use, PET and PE release carcinogenic substances. PA is not easy to digest after entering an organism and readily produces nausea, vomiting, and other symptoms. PVC contains chlorine, which is released into the environment under high temperatures and ultraviolet radiation, polluting the environment. Although the content of microplastics in the urban rivers of Chengdu is not high, we should keep monitoring the content and types of microplastics in these rivers.

## 4. Conclusions

We studied the microplastics pollution status in four urban rivers in Chengdu, namely, the Jinma River, Fuhe, Shahe, and South River, and drew the following conclusions:

(1) The urban rivers in Chengdu contained microplastics, but compared with those of cities in other regions, the content is not high, ranging from medium to low levels. There were many factors affecting the distribution of microplastic particles in water ecological systems, but through comprehensive analyses, we concluded that city population density and regional economic development had a considerable influence on concentration changes. Furthermore, by analyzing the Pearson coefficient correlation of plastic granules quantity with urban population and economic development capacity, the microplastic content in the urban rivers was significantly correlated with the population size, which confirmed our hypotheses. Pollution sources of microplastics in the urban rivers are very complex and difficult to determine. Therefore, phenomena such as municipal pipe network overflow, direct discharge of sewage, and rainwater runoff pollution may critically impact the number of microplastics in rivers.
(2) The forms of microplastics in the studied rivers are mainly debris, particles, and fibers. The microplastics less than 500 μm in size constitute a proportion as high as 69.8%, and most of the microplastics were transparent in color. Small plastic particles are easily eaten by aquatic biota in studied rivers and birds foraging nearby, which may be harmful. Microplastics concentrated in fish and shrimp are transmitted through the food web and may pose a threat to human health.
(3) PET and PA were the main types of microplastics in the rivers, accounting for 52.5% and 20.8% of the total microplastics in all samples, respectively. EVOH, commonly used in food packaging, accounts for 9.12%, and this type of pollution should be monitored.

Our research on microplastics is in its infancy, and there is still much work to be undertaken. For example, improving the removal efficiency of microplastic particles in sewage treatment plants; reducing their discharge to natural water bodies; studying the interaction of plastic granules with heavy metals, persistent organic pollutants, and organisms; better understanding the environmental pollution behavior of microplastics; and the enrichment of microplastics in animals and related toxicological studies will all contribute to understanding the impact of microplastics on human health. For the sake of a healthier and safer ecological environment for human beings, we recommend that all relevant departments establish pollution emission and evaluation standards for microplastics.

## Figures and Tables

**Table 1:** Pollution abundance of microplastics in surface water in different areas.

No.	Location	Size Class (mm)	Mean Concentration of Microplastic, Items/L	Dominant Microplastic	Reference
1	Los Angeles River, USA	1~4.5	12.9	--	[15]
2	San Gabriel River, USA	1~4.5	0.41	--	[15]
3	Pearl River, China	0. 02~5	0.38~7.92	PE, PP	[16]
4	Saigon River, Vietnam	0.2~1	172 ± 519	--	[21]
5	urban surface waters of Wuhan, China	0.05~5	0.921~10.516	PET, PP	[22]
6	Taihu Lake, China	0.3~5	3.4~25.8	PE	[27]
7	urban rivers in Chengdu, China	0. 02~5	5.00~10.5	PET, PA	This study

**Table 2:** The correlation coefficients of population and GDP with concentration (GDP: gross domestic product. GDP is an important indicator to measure the economic status and development level of a country or region.).

Item	Concentration, Items/L	Population, Million	GDP, Billion
Shahe	5	264.73	162.8
South river	7.5	265.38	146.3
Jinma river	9.83	277.73	101.4
Fuhe	10.5	702.47	351.1
Pearson coefficient	1	0.98 *	0.5

* *p* < 0.05.

## Data Availability

The datasets generated during and/or analyzed during the current study are available from the corresponding author on reasonable request.
